# An enhanced state convergence architecture incorporating disturbance observer for bilateral teleoperation systems

**DOI:** 10.1177/1729881419880052

**Published:** 2019-10-09

**Authors:** Muhammad Usman Asad, Umar Farooq, Jason Gu, Valentina E Balas, Ghulam Abbas, Marius Balas, Vlad Muresan

**Affiliations:** 1 Department of Electrical and Computer Engineering, Dalhousie University Halifax, Nova Scotia, Canada; 2 Department of Electrical Engineering, University of the Punjab, Lahore, Pakistan; 3 College of Electrical and Information Engineering, Lanzhou University of Technology, Lanzhou, China; 4 Department of Automation and Applied Informatics, Aurel Vlaicu University of Arad, Arad, Romania; 5 Department of Electrical Engineering, The University of Lahore, Lahore, Pakistan; 6 Faculty of Automation and Computer Sciences, Technical University of Cluj-Napoca, Cluj-Napoca, Romania

**Keywords:** Control in telerobotics, robot manipulation and control, haptics, robot arms and manipulators, robot manipulation and control, autonomous control, robot manipulation and control, human robot interaction, service robotics, force sensors, robot sensors and sensor networks, sensor signal processing, robot sensors and sensor networks

## Abstract

To bilaterally control an *n*th-order teleoperation system modeled on state space, state convergence methodology provides an elegant way to design control gains through a solution of 3*n* + 1 equations. These design conditions are obtained by allowing the master–slave error to evolve as an autonomous system and then assigning the desired dynamic behavior to the slave and error systems. The controller, thus obtained, ensures the motion synchronization of master and slave systems with adjustable force reflection to the operator. Although simple to design and easy to implement, state convergence method suffers from its dependence on model parameters, and thus the performance of the controller may degrade in the presence of parametric uncertainties. To address this limitation, we propose to integrate an extended state observer in the existing state convergence architecture which will not only compensate the modeling inaccuracies by treating them as a disturbance but will also provide the estimates of the master and slave states. These estimated states are then used to construct the bilateral controller which is designed by following the method of state convergence. In this case, 2*n* + 2 additional design equations are required to be solved to fix the observer gains. To validate the proposed enhancement in the state convergence architecture, simulations and semi-real-time experiments are performed in MATLAB/Simulink environment on a single degree-of-freedom teleoperation system.

## Introduction

A bilateral teleoperation system extends the human capability to perform a task at a distant location through the use of robotic devices. The task is initiated by the human operator by driving the master robotic system. The trajectory information of the master robotic system is then transmitted over a communication channel to the remote site where a slave robotic system is installed to perform the intended task. While executing the task, slave robotic system transmits environment-related information back to the master robotic system so that human operator remains aware of the events happening at the remote location. This bidirectional flow of information between the local and remote sites is necessary for the successful completion of the task. At the same time, this bilateral connection poses challenges for the designers due to the presence of time delays, system uncertainties, and bandwidth constraints.^1^

The problem of time delay in bilateral teleoperation systems has been extensively addressed in the literature due to its negative effect on the stability of such systems. A class of control algorithms, known as passivity schemes, has emerged based on the concepts of transmission line theory which avoids the destabilization of bilateral teleoperation systems by encoding the system’s variables into wave-variables and transmitting them across the communication channel.^2,3^ The tuning of these wave-variable controllers for optimum performance is also discussed based on linear control theory.^4^ The over-dissipation phenomenon of the passivity controllers is also addressed and passivity observers are proposed to activate the passivity controllers upon the detection of active energy.^5^ To deal with system uncertainties, passivity schemes are combined with neural networks.^6,7^ Other popular techniques for bilateral teleoperation systems include H-∞ control,^8,9^ adaptive control,^10–12^ fuzzy model-based control,^13^ sliding mode control,^14^ model-mediated control,^15–17^ disturbance observer,^18–21^ and hybrid schemes.^22–24^ More recently, prescribed performance method is employed to guarantee the desired transient behavior of bilateral teleoperation systems.^25,26^

State convergence is another novel method for contact motion of bilateral teleoperation systems.^27–29^ It models *n*th-order master and slave systems on state space and provides a systematic design procedure to establish the bilateral communication between these systems. The control gains of the scheme are obtained by solving 3*n* + 1 design equations which are the result of (1) transforming slave–master error into an autonomous system and (2) imposing the desired dynamic behavior to the slave and error systems. This model-based scheme is applicable to linear teleoperation systems having small constant time delay in the communication channel. To minimize the reliance of state convergence scheme on model parameters, this study proposes to employ disturbance observers for estimating the uncertainties in the master and slave systems by treating them as disturbances. The particular disturbance observer used here is known as extended state observer. The choice of this disturbance estimator over others is made due to the assumption of slowly varying disturbances.^30–35^ The estimated states are used to construct bilateral control law which is designed by following the method of state convergence such that the stability of the closed-loop teleoperation system is also ensured. The proposed scheme is finally validated in MATLAB/Simulink (version R2014a) environment on a single degree-of-freedom nonlinear time-delayed teleoperation system. It is important to mention that linear disturbance observers have been used by the researchers to control teleoperation systems in oblique coordinates^19^ and modal spaces.^20^ In addition, the use of nonlinear disturbance observers has also been reported.^21^ However, to the best of authors’ knowledge, disturbance observer-based state convergence scheme has not yet been discussed in the literature which has motivated us to perform this study. The proposal enhances the state convergence architecture in two ways: (1) extending the operation from linear to nonlinear systems and (2) estimation of master and slave systems’ states for use in bilateral laws.

This article is structured as follows: State convergence architecture is reviewed in the second section. Proposed enhancement in state convergence architecture is explained in the third section. Results are presented in the fourth section, while conclusions are drawn in the fifth section.

## State convergence architecture

**Figure 1.**
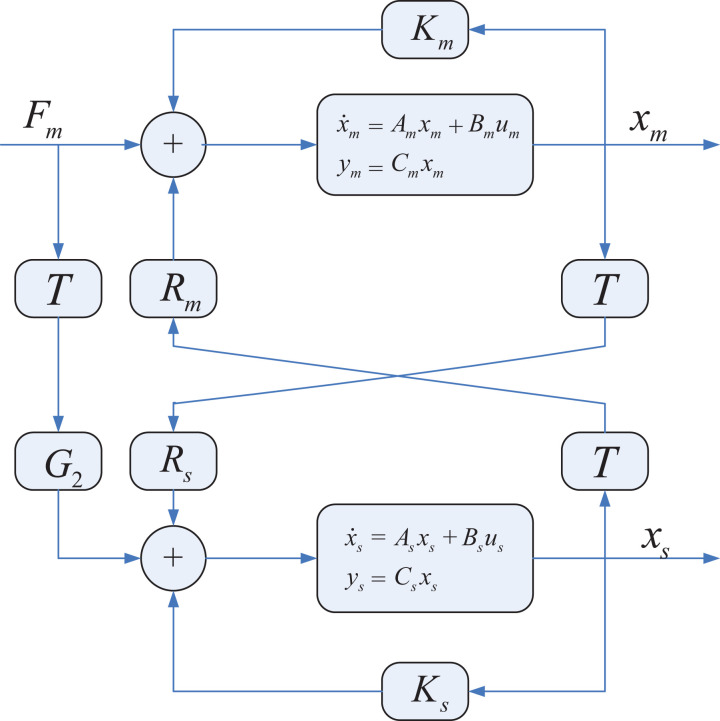
State convergence architecture.^27^

State convergence architecture,^27^ shown in Figure 1, establishes a bilateral connection between the master and slave systems which can be represented by *n*th-order linear differential equation and modeled on state space as


1 $$\begin{array}{l} {\dot{x}}_{z}=A_{z}x_{z}+B_{z}u_{z}\\ y_{z}=C_{z}x_{z} \end{array}$$

where subscript “*z*” is to be replaced with “*m*” for the master system and with “*s*” for the slave system. Various matrix entries in equation (1) are given as


2 $$\begin{array}{l} A_{z}=\left[\begin{array}{ccccc} 0 & 1 & 0 & \cdots & 0\\ 0 & 0 & 1 & \cdots & 0\\ & & \vdots & & \\ 0 & 0 & 0 & \cdots & 1\\ -a_{z0} & -a_{z1} & -a_{z2} & \cdots & -a_{zn-1} \end{array}\right],B_{z}=\left[\begin{array}{c} 0\\ 0\\ \vdots \\ 0\\ b_{z0} \end{array}\right]\\ C_{z}=\left[\begin{array}{ccccc} 1 & 0 & 0 & \cdots & 0 \end{array}\right] \end{array}$$

Various parameters forming the state convergence architecture are defined as $K_{m}=\left[\begin{array}{cccc} k_{m1} & k_{m2} & \cdots & k_{mn} \end{array}\right]$ is the feedback stabilizing controller for master system, $K_{s}=\left[\begin{array}{cccc} k_{s1}^{*}+k_{e} & k_{s2}^{*}+b_{e} & \cdots & k_{sn} \end{array}\right]$ is the feedback stabilizing controller for the slave system which also includes the stiffness (*k_e_*) and damping (*b_e_*) terms of the environment to counter the environmental force, $R_{m}=\left[\begin{array}{cccc} k_{f}k_{e} & k_{f}b_{e} & \cdots & 0 \end{array}\right]$ transfers the scaled effect of slave’s motion to the master system as the slave interacts with the environment where scaling is achieved through the force feedback gain (*k_f_*), $R_{s}=\left[\begin{array}{cccc} r_{s1} & r_{s2} & \cdots & r_{sn} \end{array}\right]$ transfers the effect of master’s motion to the slave system, and $G_{2}$ is the force transmission gain from the master to the slave system when the operator exerts a force (*F_m_*) to move the master system. Of these parameters, $G_{2},K_{m},K_{s},R_{s}$ are unknown and found through a solution of 3*n* + 1 design conditions, as described in the study by Azorin et al.^27^

### Remark 1

State convergence scheme employs the model parameters in equation (2) for the design of control gains. In practice, parametric uncertainties cannot be avoided and may degrade the performance of the controller. The effect of these uncertainties has been numerically evaluated on the performance of the state convergence controller in the literature.^28,29^ It is found that the bilateral controller is quite robust to more than 50% variation in the model parameters. However, the effect of these parametric uncertainties has not been explicitly considered during the design phase of the scheme which has motivated us to perform this study. Note that operator and environmental force estimation will not be undertaken in this study.

## Proposed enhanced state convergence architecture

**Figure 2.**
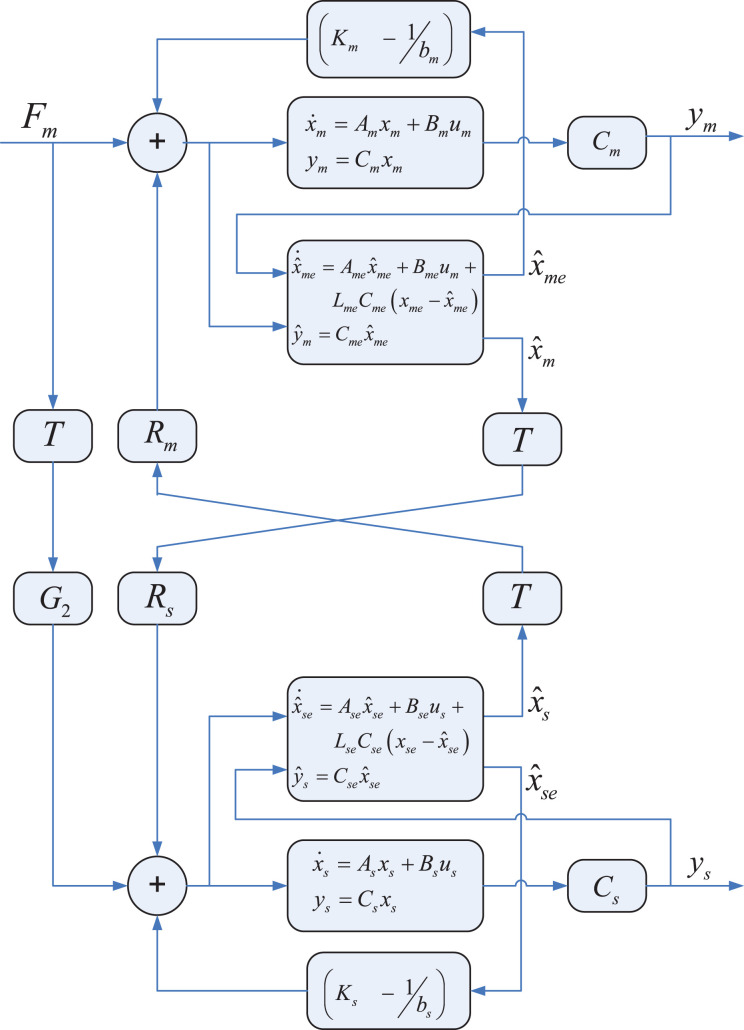
State convergence architecture incorporating disturbance observer.

To deal with uncertainties, we propose an enhanced version of the state convergence architecture, shown in Figure 2, where extended state observers are used to estimate the uncertainties present in the master and slave systems. These observers also provide the estimates of the master and slave systems’ states. The disturbance and state estimates are then used to form the bilateral control law. We proceed by considering the following nonlinear model of the master (*z* = *m*) and slave (*z* = *s*) systems


3 $$\begin{array}{l} {\dot{x}}_{z1}=x_{z2}\\ {\dot{x}}_{z2}=x_{z3}\\ \vdots \\ {\dot{x}}_{zn}=f_{z}\left(x_{z}\right)+b_{z}u_{z}\\ y_{z}=x_{z1} \end{array}$$

In equation (3), $f_{z}$ is considered to be completely unknown and will be estimated using the disturbance observer along with other system’s states. To this end, we first rewrite equation (3) by considering the disturbance $d_{z}=f_{z}$ as an additional (*n* + 1)th-state


4 $$\begin{array}{l} {\dot{x}}_{ze1}=x_{ze2}\\ {\dot{x}}_{ze2}=x_{ze3}\\ \vdots \\ {\dot{x}}_{zen}=x_{ze\left(n+1\right)}+b_{z}u_{z}\\ {\dot{x}}_{ze\left(n+1\right)}=h_{z}\\ y_{z}=x_{ze1} \end{array}$$

Note the slight change of notation in equation (4) where subscript “*e*” is added to denote the extended system. Further, time derivative of the disturbance also appears in equation (10), that is, $h_{z}={\dot{d}}_{z}$ . Before further development, we write systems (3) and (4) in compact form as


5 $$\begin{array}{l} {\dot{x}}_{z}=A_{z}x_{z}+B_{z}u_{z}+E_{z}d_{z}\\ y_{z}=C_{z}x_{z} \end{array}$$

6 $$\begin{array}{l} A_{z}=\left[\begin{array}{ccccc} 0 & 1 & 0 & \cdots & 0\\ 0 & 0 & 1 & \cdots & 0\\ & & \vdots & & \\ 0 & 0 & 0 & \cdots & 1\\ 0 & 0 & 0 & \cdots & 0 \end{array}\right],\;B_{z}=\left[\begin{array}{c} 0\\ 0\\ \vdots \\ 0\\ b_{z} \end{array}\right],\;E_{z}=\left[\begin{array}{c} 0\\ 0\\ \vdots \\ 0\\ 1 \end{array}\right]\\ C_{z}=\left[\begin{array}{ccccc} 1 & 0 & 0 & \cdots & 0 \end{array}\right] \end{array}$$

7 $$\begin{array}{l} {\dot{x}}_{ze}=A_{ze}x_{ze}+B_{ze}u_{ze}+E_{ze}h_{z}\\ y_{z}=C_{ze}x_{ze} \end{array}$$

8 $$\begin{array}{l} A_{ze}=\left[\begin{array}{cccccc} 0 & 1 & 0 & \cdots & 0 & 0\\ 0 & 0 & 1 & \cdots & 0 & 0\\ & & & \vdots & & \\ 0 & 0 & 0 & \cdots & 1 & 0\\ 0 & 0 & 0 & \cdots & 0 & 1\\ 0 & 0 & 0 & \cdots & 0 & 0 \end{array}\right],\;B_{ze}=\left[\begin{array}{c} 0\\ 0\\ \vdots \\ 0\\ b_{z}\\ 0 \end{array}\right],\;E_{ze}=\left[\begin{array}{c} 0\\ 0\\ \vdots \\ 0\\ 0\\ 1 \end{array}\right]\\ C_{ze}=\left[\begin{array}{cccccc} 1 & 0 & 0 & \cdots & 0 & 0 \end{array}\right] \end{array}$$

Considering the virtual extended system of equation (7), we construct the real extended observer as


9 $$\begin{array}{l} {\dot{\hat{x}}}_{ze}=A_{ze}{\hat{x}}_{ze}+B_{ze}u_{z}+L_{ze}C_{ze}\left(x_{ze}-{\hat{x}}_{ze}\right)\\ {\hat{y}}_{z}=C_{ze}{\hat{x}}_{ze} \end{array}$$

In equation (9), ${\hat{x}}_{ze}$ are the estimated states and $L_{ze}$ is the observer gain given as $L_{ze}={\left[\begin{array}{cccccc} l_{ze1} & l_{ze2} & l_{ze3} & \cdots & l_{zen} & l_{ze\left(n+1\right)} \end{array}\right]}^{\text{T}}$ . Let us define the observer error for the master and slave system as


10 $$e_{zo}=x_{ze}-{\hat{x}}_{ze}$$

Observer error dynamics can now be written using equations (7), (9), and (10) as


11 $${\dot{e}}_{zo}=\left(A_{ze}-L_{ze}C_{ze}\right)e_{zo}+E_{ze}h_{z}$$

We now construct the bilateral state convergence controller using estimated states as


12 $$u_{m}=-\frac{1}{b_{m}}{\hat{x}}_{me\left(n+1\right)}+K_{m}{\hat{x}}_{m}+R_{m}{\hat{x}}_{s}\left(t-T\right)+F_{m}$$

13 $$u_{s}=-\frac{1}{b_{s}}{\hat{x}}_{se\left(n+1\right)}+K_{s}{\hat{x}}_{s}+R_{s}{\hat{x}}_{m}\left(t-T\right)+G_{2}F_{m}\left(t-T\right)$$

By plugging equations (12) and (13) in equation (5) and using equations (10) and (11) and noting Bzbz=Ez , closed-loop augmented slave–master system is found to be


14 $$\begin{array}{c} \left[\begin{array}{cc} I & A_{12t}\\ A_{21t} & I \end{array}\right]\left[\begin{array}{c} {\dot{x}}_{s}\\ {\dot{x}}_{m} \end{array}\right]=\left[\begin{array}{cc} A_{11a} & A_{12a}\\ A_{21a} & A_{22a} \end{array}\right]\left[\begin{array}{c} x_{s}\\ x_{m} \end{array}\right]\\ +\left[\begin{array}{cc} A_{11e} & A_{12e}\\ A_{21e} & A_{22e} \end{array}\right]\left[\begin{array}{c} e_{so}\\ e_{mo} \end{array}\right]\\ \text{+}\left[\begin{array}{cc} 0 & A_{12h}\\ A_{21h} & 0 \end{array}\right]\left[\begin{array}{c} h_{s}\\ h_{m} \end{array}\right]+\left[\begin{array}{c} B_{1}\\ B_{2} \end{array}\right]F_{m} \end{array}$$

where $A_{11a}=A_{s}+B_{s}K_{s}$ , $A_{12a}=B_{s}R_{s}$ , $A_{21a}=B_{m}R_{m}$ , $A_{22a}$ $=A_{m}+B_{m}K_{m}$ , $A_{11e}=-\left(\begin{array}{cc} B_{s}K_{s} & -E_{s} \end{array}\right)$ , $A_{12e}=\left(\begin{array}{cc} TB_{s}R_{s} & 0 \end{array}\right)$ $\left(A_{me}-L_{me}C_{me}\right)-\left(\begin{array}{cc} B_{s}R_{s} & 0 \end{array}\right)$ , $A_{21e}=\left(\begin{array}{cc} TB_{m}R_{m} & 0 \end{array}\right)(A_{se}-L_{se}$ $C_{se})-\left(\begin{array}{cc} B_{m}R_{m} & 0 \end{array}\right)$ , $A_{22e}=-\left(\begin{array}{cc} B_{m}K_{m} & -E_{m} \end{array}\right)$ , $A_{12t}=TB_{s}R_{s}$ , $A_{21t}=TB_{m}R_{m}$ , $A_{12h}=\left(\begin{array}{cc} TB_{s}R_{s} & 0 \end{array}\right)E_{me}$ , $A_{21h}=\left(\begin{array}{cc} TB_{m}R_{m} & 0 \end{array}\right)E_{se}$ , $B_{1}=B_{s}G_{2}$ , and $B_{2}=B_{m}$ . By pre-multiplying equation (14) with the inverse of matrix $\left[\begin{array}{ccccc} I & A_{12t} & ; & A_{21t} & I \end{array}\right]$ and combining the resulting expression with the observer dynamics (11), we have


15 $$\left[\begin{array}{c} {\dot{x}}_{s}\\ {\dot{x}}_{m}\\ {\dot{e}}_{so}\\ {\dot{e}}_{mo} \end{array}\right]=\left[\begin{array}{cccc} A_{11} & A_{12} & A_{13} & A_{14}\\ A_{21} & A_{22} & A_{23} & A_{24}\\ 0 & 0 & A_{34} & 0\\ 0 & 0 & 0 & A_{44} \end{array}\right]\left[\begin{array}{c} x_{s}\\ x_{m}\\ e_{so}\\ e_{mo} \end{array}\right]+\left[\begin{array}{c} B_{11}\\ B_{21}\\ 0\\ 0 \end{array}\right]F_{m}+\left[\begin{array}{cc} E_{11} & E_{12}\\ E_{21} & E_{22}\\ E_{31} & 0\\ 0 & E_{42} \end{array}\right]\left[\begin{array}{c} h_{s}\\ h_{m} \end{array}\right]$$

where $A_{11}=A_{i1}A_{11a}+A_{i2}A_{21a}$ , $A_{12}=A_{i1}A_{12a}+A_{i2}A_{22a}$ , $A_{13}=A_{i1}A_{11e}+A_{i2}A_{21e}$ , $A_{14}=A_{i1}A_{12e}+A_{i2}A_{22e}$ , $A_{21}=A_{i3}A_{11a}+A_{i4}A_{12a}$ , $A_{22}=A_{i3}A_{12a}+A_{i4}A_{22a}$ , $A_{23}=A_{i3}A_{11e}+A_{i4}A_{21e}$ , $A_{24}=A_{i3}A_{12e}+A_{i4}A_{22e}$ , $A_{34}=A_{se}-L_{se}C_{se}$ , $A_{44}=A_{me}-L_{me}C_{me}$ , $B_{11}=A_{i1}B_{1}+A_{i2}B_{2}$ , $B_{21}=A_{i3}B_{1}+A_{i4}B_{2}$ , $E_{11}=A_{i2}A_{21h}$ , $E_{12}=A_{i1}A_{12h}$ , $E_{21}=A_{i4}A_{21h}$ , $E_{22}=A_{i3}A_{12h}$ , $E_{31}=E_{se}$ , $E_{42}=E_{me}$ , $A_{i1}=I+A_{12t}\Xi A_{21t}$ , $A_{i2}=-A_{12t}\Xi$ , $A_{i3}=-\Xi A_{21t}$ , $A_{i4}=\Xi$ , $\Xi =(I-{A_{21t}A_{12t})}^{-1}$ . Following the method of state convergence, we replace the master system in equation (15) with the slave–master error system. To achieve this, we introduce the following linear transformation


16 $$\left[\begin{array}{c} x_{s}\\ x_{e}\\ e_{so}\\ e_{mo} \end{array}\right]=\left[\begin{array}{cccc} I & 0 & 0 & 0\\ I & -I & 0 & 0\\ 0 & 0 & I & 0\\ 0 & 0 & 0 & I \end{array}\right]\left[\begin{array}{c} x_{s}\\ x_{m}\\ e_{so}\\ e_{mo} \end{array}\right]$$

The time derivative of equation (16) in combination with equation (15) yields the following augmented system


17 $$\left[\begin{array}{c} {\dot{x}}_{s}\\ {\dot{x}}_{e}\\ {\dot{e}}_{so}\\ {\dot{e}}_{mo} \end{array}\right]=\left[\begin{array}{cccc} {\tilde{A}}_{11} & {\tilde{A}}_{12} & {\tilde{A}}_{13} & {\tilde{A}}_{14}\\ {\tilde{A}}_{21} & {\tilde{A}}_{22} & {\tilde{A}}_{23} & {\tilde{A}}_{24}\\ 0 & 0 & {\tilde{A}}_{34} & 0\\ 0 & 0 & 0 & {\tilde{A}}_{44} \end{array}\right]\left[\begin{array}{c} x_{s}\\ x_{e}\\ e_{so}\\ e_{mo} \end{array}\right]+\left[\begin{array}{c} {\tilde{B}}_{11}\\ {\tilde{B}}_{21}\\ 0\\ 0 \end{array}\right]F_{m}+\left[\begin{array}{cc} {\tilde{E}}_{11} & {\tilde{E}}_{12}\\ {\tilde{E}}_{21} & {\tilde{E}}_{12}\\ {\tilde{E}}_{31} & 0\\ 0 & {\tilde{E}}_{42} \end{array}\right]\left[\begin{array}{c} h_{s}\\ h_{m} \end{array}\right]$$

where ${\tilde{A}}_{11}=A_{11}+A_{12}$ , ${\tilde{A}}_{12}=-A_{12}$ , ${\tilde{A}}_{13}=A_{13}$ , ${\tilde{A}}_{14}=A_{14}$ , ${\tilde{A}}_{21}=A_{11}-A_{21}+A_{12}-A_{22}$ , ${\tilde{A}}_{22}=-A_{12}+A_{22}$ , ${\tilde{A}}_{23}=A_{13}-A_{23}$ , ${\tilde{A}}_{24}=A_{14}-A_{24}$ , ${\tilde{A}}_{33}=A_{33}$ , ${\tilde{A}}_{44}=A_{44}$ , ${\tilde{B}}_{11}=B_{11}$ , ${\tilde{B}}_{21}=B_{11}-B_{21}$ , ${\tilde{E}}_{11}=E_{11}$ , ${\tilde{E}}_{12}=E_{12}$ , ${\tilde{E}}_{21}=E_{11}-E_{21}$ , ${\tilde{E}}_{22}=E_{12}-E_{22}$ , ${\tilde{E}}_{31}=E_{31}$ , ${\tilde{E}}_{42}=E_{42}$ . By eliminating the effect of slave system’s states and operator’s force on the error system in equation (17), we obtain the following *n* + 1 design conditions


18 $${\tilde{A}}_{21}=0,{\tilde{B}}_{21}=0$$

Note that the effect of observers’ errors is ignored on the slave–master error system as fast dynamic behavior will be assigned to the observers. Further, effect of disturbance terms is not considered in the design phase which can be associated with slow varying nature of the disturbance.^32–34^ Now, by comparing the characteristic polynomial of the augmented system in equation (17) with the desired polynomials, we have


19 $$\begin{array}{l} \left|sI-{\tilde{A}}_{11}\right|=s^{n}+p_{n-1}s^{n-1}+\cdots +p_{1}s+p_{0}\\ \left|sI-{\tilde{A}}_{22}\right|=s^{n}+q_{n-1}s^{n-1}+\cdots +q_{1}s+q_{0}\\ \left|sI-{\tilde{A}}_{34}\right|=s^{n}+r_{n-1}s^{n-1}+\cdots +r_{1}s+r_{0}\\ \left|sI-{\tilde{A}}_{44}\right|=s^{n}+w_{n-1}s^{n-1}+\cdots +w_{1}s+w_{0} \end{array}$$

In equation (19), $p_{i},q_{i},r_{i},w_{i}$ are coefficients of the desired polynomials for the slave, error, slave observer, and master observer systems, respectively. Equations (18) and (19) form together a set of 5*n* + 3 design conditions which can be solved to find 3*n* + 1 unknown controller gains $\left(G_{2},K_{m},K_{s},R_{s}\right)$ and 2*n* + 2 unknown observer $\left(L_{se},L_{me}\right)$ gains of the enhanced state convergence scheme.

## Results

To validate the proposed disturbance observer-based state convergence controller, we perform simulations in MATLAB/Simulink environment by considering a single degree-of-freedom master and slave systems as ${\dot{x}}_{z1}=x_{z2}$ , ${\dot{x}}_{z2}=f_{z}\left(x_{z}\right)+u_{z}$ , $f_{z}=-a_{z1}\text{sin}\left(x_{z1}\right)-a_{z2}x_{z2}+\left(b_{z}-1\right)u_{z}$ . Thus, we have the following model for the design phase


20 $$A_{z}=\left[\begin{array}{cc} 0 & 1\\ 0 & 0 \end{array}\right],\;B_{z}=\left[\begin{array}{c} 0\\ 1 \end{array}\right],\;A_{ze}=\left[\begin{array}{ccc} 0 & 1 & 0\\ 0 & 0 & 1\\ 0 & 0 & 0 \end{array}\right],B_{ze}=\left[\begin{array}{c} 0\\ 1\\ 0 \end{array}\right]$$

We assume various parameters of the teleoperation system as $a_{m1}=1$ , $a_{s1}=0.5$ , $a_{m2}=\text{7.1429}$ , $a_{s2}=6.25$ , $b_{m}=\text{0.2656}$ , $b_{s}=\text{0.2729}$ , T=0.2 s , $k_{f}=1$ , $k_{e}=20\;\text{N}\;\text{m/rad}$ , and $b_{e}=\text{0.1}\;\text{N}\;\text{m}\;\text{s/rad}$ ^27^ Further, we select the coefficients of polynomials in equation (25) as $p_{0}=20$ , $p_{1}=15$ , $q_{0}=64$ , $q_{1}=16$ , $r_{0}=w_{0}=27,000$ , $r_{1}=w_{1}=2700$ , and $r_{2}=w_{2}=90$ . Now, by solving the design conditions (24) and (25), we obtain the controller and observer gains as


21 $$\begin{array}{l} G_{2}=1.3436\\ K_{m}=\left[\begin{array}{cc} -40.40 & -11.40 \end{array}\right]\\ K_{s}=\left[\begin{array}{cc} -15.4079 & -20.7101 \end{array}\right]\\ R_{s}=\left[\begin{array}{cc} 7.9981 & 1.8524 \end{array}\right]\\ L_{me}=L_{se}={\left[\begin{array}{ccc} 90 & 2700 & 27000 \end{array}\right]}^{\text{T}} \end{array}$$

**Figure 3.**
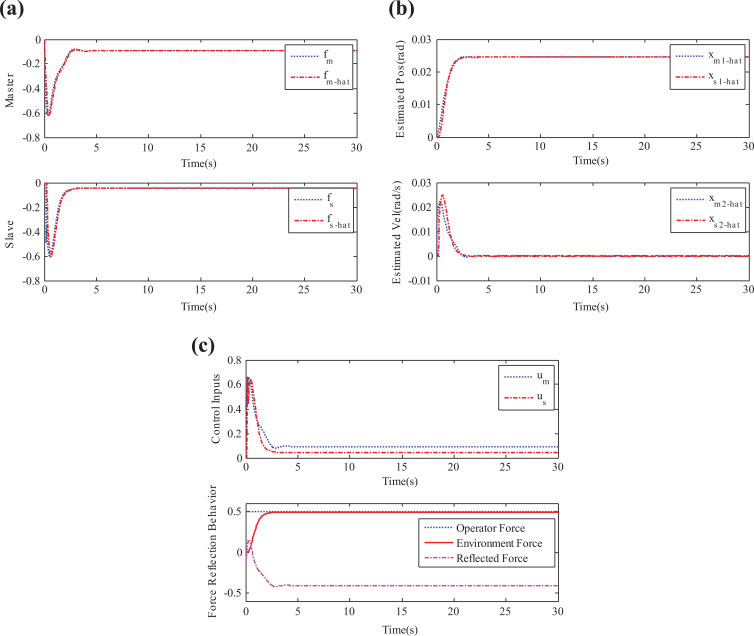
Simulation results: (a) disturbance estimation, (b) estimated position and velocity states, and (c) control torques.

Note that control gain $K_{s}$ in equation (21) contains the environment information also, as pointed out in the “State convergence architecture” section. In the absence of such information, steady-state error will exist between the master and slave positions as the slave interacts with the environment. We now perform simulations of time-delayed nonlinear teleoperation system under the control gains of equation (21), and the results are depicted in Figure 3. It can be seen that disturbance is well-estimated by the observer, and slave is following the master system. In addition, environmental force is also reflected to the operator whose amount can be adjusted through the coefficient $p_{0}$ .

To show some advantage of the proposed scheme, a comparison has been made with a recently proposed tele-controller based on radial basis function neural network (RBFNN).^36^ For the sake of completeness, we mention the RBFNN-based control laws here


22 $$\begin{array}{l} u_{z}=l_{z}s_{z}+{\hat{W}}_{z}h_{z}\left(x_{z}\right)+\eta_{z}\text{sgn}\left(s_{z}\right)-\tau_{z},\;z=m,s\\ s_{z}={\dot{e}}_{z}+k_{z}e_{z},\;z=m,s\\ {\dot{\hat{W}}}_{z}=\frac{1}{\delta_{z}}s_{z}h_{z}\left(x_{z}\right),\;z=m,s \end{array}$$

**Figure 4.**
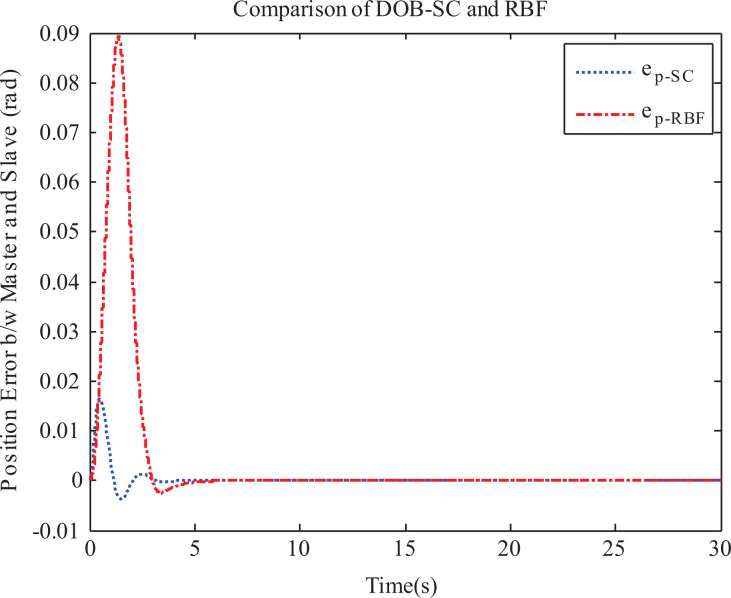
Comparison of proposed scheme and RBFNN. RBFNN: radial basis function neural network.

Using different parameters of RBFNN controllers as $\eta_{m}=\eta_{s}=0.25$ , $\delta_{m}=\delta_{m}=0.05$ , $l_{m}=l_{s}=15$ , $k_{m}=k_{s}=15$ , $\tau_{f}=0.025$ , sat=±0.05 , n=5 , $b_{j}=0.5$ , $C_{i}=-1:0.5:1$ , $M_{r}=1$ , $C_{r}=4$ , and $G_{r}=4$ , we simulate the same time-delayed tele-robotic system under the control of equation (22), and various states are recorded. Figure 4 depicts the position error between the master and slave systems as yielded by both the proposed and RBFNN controllers. It can be seen that the proposed controller offers better transient performance as compared to RBFNN controller for the same final master position. However, the proposed controller is only valid for the constant time delays.

**Figure 5.**
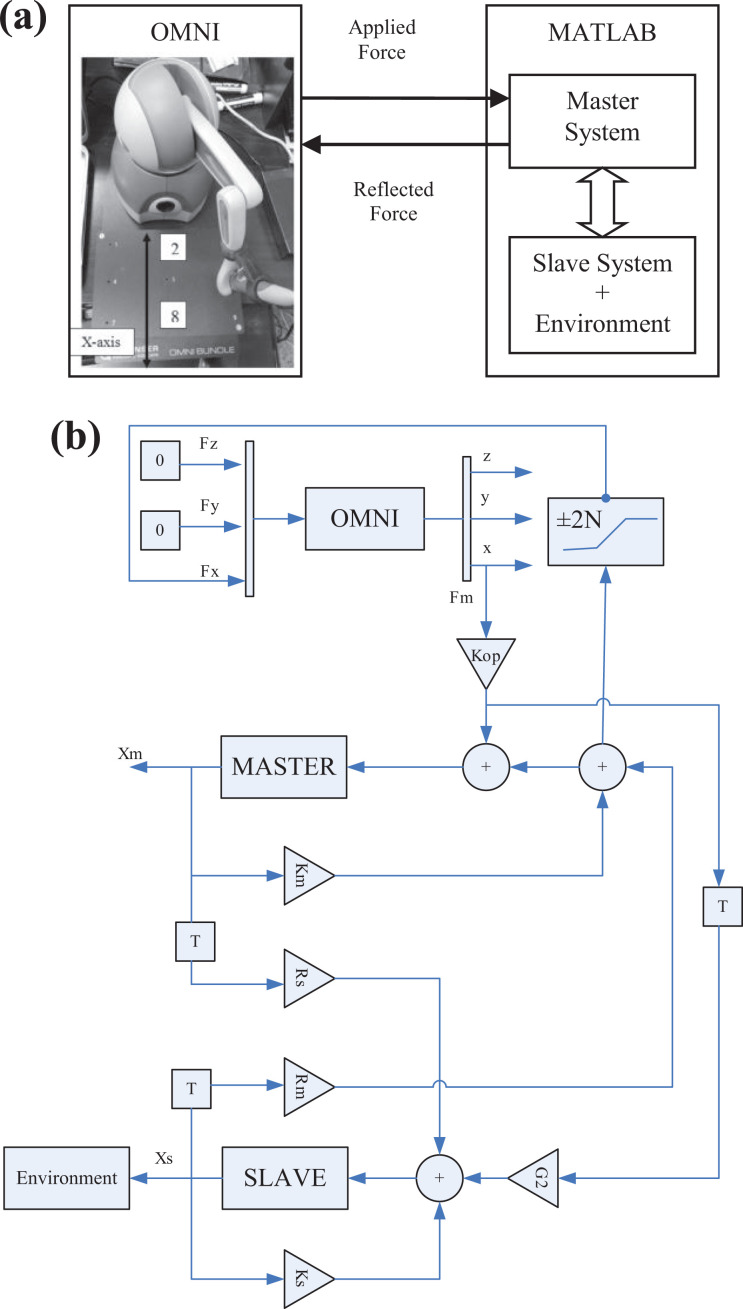
Experimental framework: (a) layout and (b) more detailed view (observer part is not shown for simplicity).

**Figure 6.**
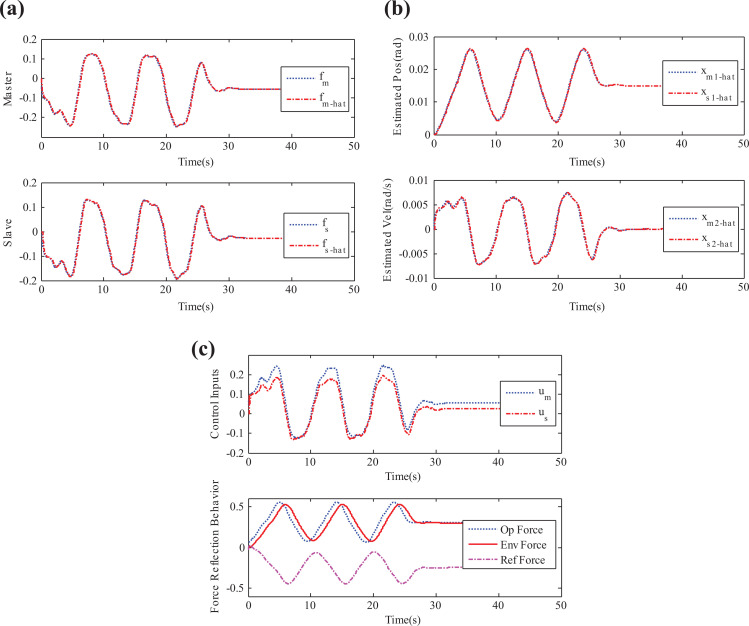
Experimental results: (a) disturbance estimation, (b) estimated position and velocity states, and (c) control torques.

Finally, we perform some semi-real-time experiments in QUARC/Simulink (version v2.5.1062) environment using the geomagic haptic device. To this end, haptic device is operated along the *x*-axis to generate a time-varying force for the teleoperation system running in Simulink environment. This force is governed by the relation $F_{m}\left(t\right)=k_{op}\left(x_{op}\left(t\right)-x_{0}\right)$ . Here, scaling factor is assumed to be $k_{\text{op}}=5$ , while operator’s position $\left(x_{\text{op}}\right)$ lies in the range [0.1,0.2] which corresponds to the motion of the link between points “2” and “8” on the cardboard, as shown in Figure 5. Further, Simulink model is designed such that the reflected force, as generated by the proposed controller, is also directed to the haptic device. In this way, loop is closed around the operator as he will be able to feel the slave’s interaction with the environment. Now, using the control gains of equation (21), nonlinear time-delayed teleoperation system is run under the control of haptic device and the results are shown in Figure 6. It can be seen that observer has remained successful in estimating the disturbance, and observer-based controller has established the convergence of master and slave states. Further, environmental force is also felt by the operator. These results suggest that the proposed methodology has indeed enhanced the capability of state convergence architecture to deal with uncertain nonlinear teleoperation systems.

## Conclusion

This article has presented an enhancement in the state convergence architecture for bilateral teleoperation systems through the use of disturbance observers. The proposal suggests treating uncertainties in the master and slave systems as disturbances and employing extended state observers to estimate them. State convergence control laws are then updated with these estimates. Closed-loop stability of the teleoperation system is finally established by following the method of state convergence. To validate the proposal, simulations and semi-real-time experiments are also performed in MATLAB/Simulink environment by considering a single degree-of-freedom nonlinear time-delayed teleoperation system. Future work involves designing and integrating the operator and environment estimation laws in the proposed framework with the consideration of time-varying delays of the communication channel.
